# ER Stress and Apoptosis: A New Mechanism for Retinal Cell Death

**DOI:** 10.1155/2012/589589

**Published:** 2011-12-14

**Authors:** Guangjun Jing, Joshua J. Wang, Sarah X. Zhang

**Affiliations:** ^1^Department of Medicine, Endocrinology and Diabetes, University of Oklahoma Health Sciences Center, 941 Stanton L. Young Blvd., Oklahoma City, OK 73104, USA; ^2^Harold Hamm Diabetes Center, University of Oklahoma Health Sciences Center, 941 Stanton L. Young Blvd., Oklahoma City, OK 73104, USA; ^3^Oklahoma Center for Neuroscience, University of Oklahoma Health Sciences Center, 941 Stanton L. Young Blvd., Oklahoma City, OK 73104, USA

## Abstract

The endoplasmic reticulum (ER) is the primary subcellular organelle where proteins are synthesized and folded. When the homeostasis of the ER is disturbed, unfolded or misfolded proteins accumulate in the ER lumen, resulting in ER stress. In response to ER stress, cells activate a set of tightly controlled regulatory programs, known as the unfolded protein response (UPR), to restore the normal function of the ER. However, if ER stress is sustained and the adaptive UPR fails to eliminate unfolded/misfolded proteins, apoptosis will occur to remove the stressed cells. In recent years, a large body of studies has shown that ER stress-induced apoptosis is implicated in numerous human diseases, such as diabetes and neurogenerative diseases. Moreover, emerging evidence supports a role of ER stress in retinal apoptosis and cell death in blinding disorders such as age-related macular degeneration and diabetic retinopathy. In the present review, we summarize recent progress on ER stress and apoptosis in retinal diseases, focusing on various proapoptotic and antiapoptotic pathways that are activated by the UPR, and discuss how these pathways contribute to ER stress-induced apoptosis in retinal cells.

## 1. Introduction

Retinal cell death has been widely held as a central event that leads to retinal neurodegeneration, vascular dysfunction, and eventually irreversible blindness in ocular diseases, such as glaucoma, retinal degeneration, diabetic retinopathy, and uveitis. Loss of retinal ganglion cells is considered to be a direct cause of vision loss in experimental glaucoma [1], correlating with elevated intraocular pressure (IOP) [2]. Injury of retinal pigment epithelial (RPE) cells and photoreceptors leads to photoreceptor dysfunction and retinal degeneration [3], as seen in both inherited and acquired degenerative retinal diseases such as Stargardt's Disease [4], retinitis pigmentosa [5], and age-related macular degeneration [6]. In diabetic retinopathy, high glucose and other diabetic insults, such as oxidants, advanced glycation end products (AGEs), and inflammatory cytokines, result in neural and vascular cell death [7, 8]. In streptozotocin-induced diabetic rats, both retinal neurons and vascular cells become apoptotic soon after the onset of diabetes [9]. Inflammation-driven neural and vascular cell death is also a hallmark characteristic of uveitis, a chronic eye disease that cause vision loss [10–13]. Together, these findings support a pivotal role of cell death in the pathogenesis of retinal diseases. Apoptosis, that is, programmed cell death, is the most common form of cell death in various cell types, including retinal cells. Apoptosis is tightly controlled by a variety of signaling pathways that either promote or inhibit the apoptotic cascades. Among the most extensively studied proapoptotic factors in retinal cells are oxidative stress, mitochondrial dysfunction, inflammation, ischemia, hyperglycemia, and excitotoxicity [14–18]. Intriguingly, recent evidence suggests that disturbed protein homeostasis and endoplasmic reticulum (ER) stress also contribute to apoptosis of retinal cells [19]. Moreover, ER stress activates a large number of genes involved in the control of cell fate, including antiapoptotic and proapoptotic molecules such as Bax and Bcl-2 [20, 21]. Therefore, elucidating the role and mechanisms of ER stress in retinal cell apoptosis may provide important insight into the pathogenesis of retinal diseases and help in developing new drugs to protect retinal cells and to prevent vision loss. In the present review, we discuss the potential implication of ER stress in retinal cell apoptosis, with a primary focus on the signaling transduction pathways that link ER stress with apoptosis in general as well as specific for retinal cells.

## 2. ER Stress and the Unfolded Protein Response (UPR)

The endoplasmic reticulum (ER) is the primary intracellular organelle responsible for protein folding, maturation, and trafficking [22, 23]. The ER consists of a network of folded membranes in which secretory and most membrane proteins are synthesized, posttranslationally modified, and folded into their correct three-dimensional conformations. Only properly folded (mature) proteins can be transported to the Golgi apparatus for further processing. In addition, the ER also serves as a dynamic pool of calcium, governing the intracellular calcium homeostasis [24]. Other major functions of the ER include lipid and steroid hormone synthesis, carbohydrate metabolism, and drug detoxification. Importantly, compelling evidence indicates that the ER is one of the major machinery that senses subtle environmental changes and cellular stresses, coordinates signaling pathways, and modulates cell function and cell survival. Various physiological and pathological circumstances, such as excessive mutant proteins, viral infection, energy or nutrient deprivation, as well as alteration in the redox status, can compromise the ER capacity in protein folding, resulting in the accumulation of unfolded or misfolded proteins in the ER lumen, or ER stress. In turn, misfolded proteins aggregate to form insoluble intracellular or extracellular deposit, which is toxic to the cell. It has been demonstrated that a number of age-related diseases, such as Alzheimer's diseases; inflammatory disorders, such as diabetes; and neurodegenerative diseases, such as Parkinson's disease, are associated with the build-up of misfolded or unfolded protein aggregates [25–28]. To eliminate the toxic protein components, cells activate an adaptive mechanism that consists of a number of intracellular signaling pathways, collectively known as unfolded protein response (UPR). The UPR relieves ER stress and restores the protein homeostasis through three complementary strategies: (1) halt the generation of more unfolded proteins by suppression of protein translation; (2) induce ER-related molecular chaperones to promote refolding of the unfolded proteins, and (3) activate the ER-associated protein degradation (ERAD) system to remove the unfolded proteins.

**Figure 1:**
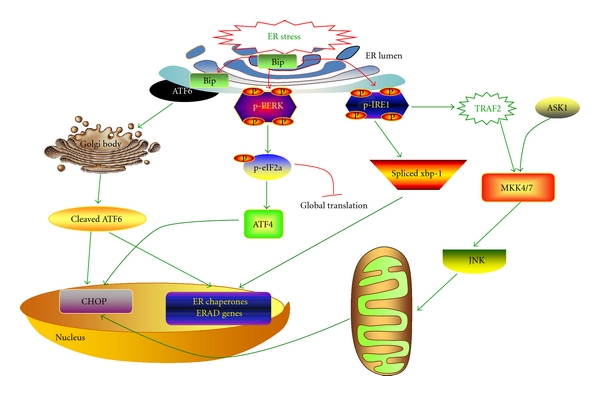
Signaling pathways of the UPR. Accumulation of unfolded proteins in ER lumen results in the ER stress. In response to ER stress, Bip dissociates from ER stress transducers and binds to unfolded and misfolded proteins, resulting in the activation of ER stress transducers- IRE1, PERK and ATF6. Upon activation, IRE1 splices the mRNA of XBP1, and produces an active transcription factor named spliced XBP1 (XBP1-S), which upregulates ER chaperones and proteins implicated in the ER-associated protein degradation (ERAD). In addition, IRE1 recruits TRAF2 and ASK1, resulting in JNK activation. The activation of PERK increases phosphorylation of eIF2*α*, leading to a global attenuation of protein synthesis and a concomitant increase in ATF4 translation. In turn, ATF4 induces CHOP, a proapoptotic transcription factor. After the dissociation of Bip, ATF6 translocates to Golgi apparatus, where it is activated by proteolysis. Activated ATF6 transcriptionally induces ERAD genes and upregulates CHOP expression.

There are three branches of UPR that are initiated by distinct ER stress transducers located on the ER membrane: PKR-like endoplasmic reticulum kinase (PERK) [29], inositol-requiring enzyme 1 (IRE1) [30, 31], and activating transcription factor 6 (ATF6) [22]. In nonstressed cells, all three ER stress transducers are kept in an inactive state through binding to the ER chaperon glucose-regulated protein 78 (Bip), which is also known as immunoglobulin binding protein (Bip) [32, 33]. Upon ER stress, excessive unfolded proteins accumulate in the ER lumen, resulting in the dissociation of GPR78 from the ER stress transducers [34], which triggers activation of the UPR branches. In eukaryotic cells, UPR is an adaptive cellular response to the disturbance of normal ER functions, which attenuate the aggregation of unfolded or misfolded proteins and promote cell survival [35]. However, during prolonged or overwhelming ER stress, UPR fails to restore the normal function of the ER, and apoptotic cascade will be activated [36, 37] (Figure 1). The exact mechanism underlying the switch of the UPR from a prosurvival mechanism to a proapoptotic response is not clear.

### 2.1. The IRE1/XBP1 Pathway

IRE1 was firstly identified as an ER transmembrane protein kinase that is essential for signaling transduction from the ER to the nucleus [38] and was subsequently found to be involved in the initiation of the UPR [39]. There are two different IRE1 proteins in mammalian cells, both of which participate in the ER stress response or UPR. IRE1*α* is ubiquitously expressed while IRE1*β* is tissue-specific [30, 40]. During ER stress, IRE1 dissociates with Bip/Bip and becomes activated. Activated IRE1 acquires the function as endogenous ribonuclease (RNase) and splices a 26-nucleotide intron from the mRNA of XBP1. The splicing results in a shift in the translational frame of the XBP1 gene, leading to the translation of a new protein, named spliced XBP1 [31, 41]. The newly generated spliced XBP1 is an active transcription factor, which in turn induces diverse downstream genes, such as ER chaperones [42] and proteins involved in ER-associated protein degradation (ERAD) [43]. These proteins work together to restore the ER homeostasis and promote cell survival. Indeed, cells deficient of XBP1 are susceptible to oxidative stress- and inflammation-induced cell death [44, 45], suggesting that XBP1-mediated adaptive UPR is an important mechanism that protects the cell from apoptosis during ER stress. In addition, the IRE1/XBP1 pathway is also essential for embryonic development. Genetic deletion of IRE1 or XBP1 is lethal to mouse embryo due to fetal liver hyperplasia [46, 47]. In addition, a recent study shows that loss of IRE1 results in severe dysfunction of the placenta and also contributes to the embryonic lethality of IRE1 KO mice [48].

### 2.2. The PERK/eIF2*α*/ATF4 Pathway

PERK is a serine/threonine protein kinase located on the ER membrane. Like IRE1, PERK is activated by ER stress via dimerization and autophosphorylation upon the dissociation with Bip. Activated PERK phosphorylates its downstream target protein, eIF2*α*, resulting in the inhibition of global protein translation [49]. However, some genes with upstream open reading frames (uORFs) in its 5′ untranslated region could escape from the eIF2*α*-initiated translational attenuation. A representative example is activating transcription factor 4 (ATF4)—human ATF4 gene contains multiple uORFs in its 5′UTR whereas the murine mRNA has two uORFs [50]. These uORFs prevent the translation of ATF4 under normal conditions but enhance its expression when eIF2*α* is phosphorylated [51, 52]. ATF4 belongs to the superfamily of DNA-binding proteins that includes the activator protein-1 (AP-1) family, cAMP-response element binding proteins (CREBs), and CREB-like proteins. As a transcription factor, ATF4 binds to the CRE site in the promoter region of target genes, inducing a battery of stress response genes involved in oxidative stress, amino acid synthesis, and transportation. In addition, ATF4 is a major inducer of C/EBP homologous protein (CHOP), which has been considered as a central mediator of ER stress-induced apoptosis. The role of CHOP in coordinating the apoptotic pathways will be discussed in detail in the following chapters.

### 2.3. The ATF6 Pathway

Besides XBP-1 and ATF4, ATF6 has been identified as another basic leucine zipper- (bZIP-) containing transcription factor induced by ER stress. ATF6 is a type II ER transmembrane protein. Like IRE1 and PERK, ATF6 binds to Bip and remains in an inactive state in unstressed cells. In response to ER stress, the Bip/ATF6 complex is dissociated, resulting in the translocation of ATF6 from ER membrane to Golgi apparatus. In Golgi apparatus, ATF6 is cleaved by two proteases, serine protease site-1 protease (S1P) and the metalloprotease site-2 protease (S2P), to produce the active form of the transcription factor [53–55]. The active ATF6 then moves to the nucleus and activates the ER stress response element- (ERSE-) related genes through binding their promoters [35]. ATF6 also regulates other URP genes, such as XBP-1 and CHOP [56].

## 3. ER Stress-Associated Apoptosis in Retina Cells

Previous studies suggest that the cell fate is dependent on the balance between the extent/severity of ER stress and the capacity of the ER to restore ER homeostasis through the UPR [57–59]. Temporal and mild ER stress can be overcome by the adaptive UPR, cell function maintained, and cells survive. However, if the stress condition is prolonged and the UPR fails to restore the ER homeostasis, the apoptotic signaling pathways will be initiated to remove the unhealthy cells. Recently, several independent studies have provided ample evidence that ER stress is a potential cause of retinal vascular and neuronal cell death in diseases such as glaucoma, diabetic retinopathy and age-related macular degeneration [19, 60–62]. ER stress has been observed in both cultured retinal cells (vascular endothelial cells, pericytes, ganglion cells, Muller cells, as well as RPE cells) and in the retina from animal models of various diseases. Not surprisingly, the role of ER stress has been extensively studied in the pathogenesis of retinitis pigmentosa (RP) with mutations of various retinal genes. In 2004, Rebello and associates reported that expression of a mutant (R14W) of carbonic anhydrase IV, a glycosylphosphatidylinositol-anchored protein that is highly expressed in the choriocapillaris of the human eye, induced upregulation of Bip, PERK, and CHOP, markers of ER stress and the unfolded protein response, accompanied by apoptosis [63]. Similarly, enhanced ER stress was reported in RP induced by the rhodopsin mutation P23H in Xenopus laevis [64] and in rats [65, 66]. Further, stimulation of the UPR in the retina or cultured retinal cells by preexposure to mild ER stress protected photoreceptor neurons from oxidative damage and cell death [67]. Moreover, overexpression of Bip, an ER chaperone that facilitates protein folding and reduces ER stress, attenuated retinal expression of CHOP and the activation of apoptotic cascade and restored retinal photoreceptor function in P23H rats [61]. In addition to the genetic models of RP, ER stress was found remarkably enhanced in retinal photoreceptors, coincident with photoreceptor cell apoptosis, in a rodent model of light damage-induced retinal degeneration [62]. These findings collectively support a causal role of ER stress in photoreceptor cell death and retinal degeneration.

Another well-studied area for ER stress-related retinal cell death is glaucoma. Increased ER stress markers were observed in retinal ganglion cells in animal models of ischemia-reperfusion and chronic glaucoma [68]. Cultured retinal ganglion cells (RGC-5, a transformed rat ganglion cell line) treated with tunicamycin, a common ER stress inducer, undergo apoptosis, accompanied by increased production of ER stress-related proteins [19, 69]. *In vivo*, intravitreal injection of tunicamycin resulted in loss of retinal ganglion cells and reduced thickness of the inner retina. Moreover, raising IOP or intravitreal injection of N-methyl-D-aspartate (NMDA), an excitotoxin that binds to the NMDA receptor and induces neuron cell death, also increased the expression of ER stress markers in retinal ganglion cells, amacrine cells, and microglial cells [19]. Pharmaceutical induction of Bip significantly attenuated tunicamycin- or NMDA-induced apoptosis in retinal ganglion cells, suggesting a pivotal role of ER stress in retinal neuron cell death [69].

Loss of retinal vascular cells and apoptosis of retinal neurons have been recognized as critical events and pathological features of diabetic retinopathy [70–72]. Although currently it remains to be investigated how ER stress signaling pathways contribute to retinal cell death induced by diabetes, recent studies by our group and others demonstrated that ER stress was induced in early stage of diabetic retinopathy and was implicated in retinal inflammation and vascular damage [73–76]. In 2009, we reported increased ER stress markers in the retina of diabetic Akita mice, in parallel with elevated expression of inflammatory genes [73]. In cultured retinal endothelial cells, ER stress was induced by hypoxia, a potent stimulator of inflammation and angiogenesis, and prevented by chemical chaperones. Moreover, we showed that induction of ER stress in the retina was sufficient to trigger an upregulation of inflammatory genes. Conversely, inhibiting ER stress protected the retina and retinal endothelial cells from inflammatory damage. In addition, activation of the adaptive UPR by preconditioning with ER stress also successfully prevented inflammatory damage to retinal endothelial cells and vascular leakage induced by diabetic stimulus [74]. These results suggest that ER stress is implicated in retinal cell damage caused by diabetes.

## 4. Signaling Pathways of ER Stress-Associated Apoptosis

**Figure 2:**
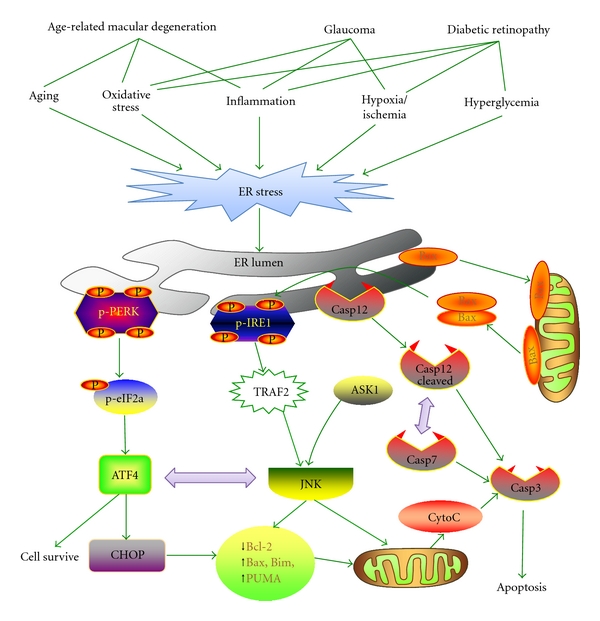
ER stress-associated apoptotic pathways in retinal diseases. A variety of pathogenic factors in chronic retinal degenerative diseases (e.g., age-related macular degeneration, glaucomatous retinopathy and diabetic retinopathy), including aging, oxidative stress, hypoxia, inflammatory factors, and hyperglycemia and others, can disturb ER function and compromise the adaptive UPR, resulting in persistent ER stress in retinal cells. This leads to sustained activation of the ATF4/CHOP pathway and the IRE1/TRAF2/ASK/JNK pathway. Both JNK and CHOP attenuate the function of the pro-survival factor Bcl-2, but enhances the activity of proapoptotic Bcl-2 proteins such as Bim, Bax, and PUMA, resulting in mitochondrial dysfunction and cytochrome *c* release. In addition, caspase-12 is activated during ER stress, which sequentially activates caspase-7 and/or caspase-3, leading to mitochondria-independent apoptosis.

It is currently unclear how ER stress induces apoptosis in various retinal cells. Generally, there are two major pathways for the initiation of apoptosis: extrinsic and intrinsic pathways [77]. The extrinsic pathway is mediated by the cell membrane death receptors. Activation of the death receptor recruits adaptor molecules and activates caspase-8 or caspase-10, which cleaves the downstream substrates, that is, other caspases including caspase-3, resulting in apoptosis [78]. The intrinsic pathway is closely related to factors anchored on the mitochondria. The insertion of these proapoptotic proteins changes the mitochondrial membrane permeability, resulting in the release of cytochrome *c* from mitochondria into the cytosol. Then cytochrome *c* binds to Apaf-1 and activates caspase-9 and then caspase-3, leading to the execution of cell death. In addition, accumulating evidence suggests that calcium release from the ER can also initiate the cell death signals, either by directly activating death receptors or by altering the sensitivity of mitochondria. Finally, these apoptotic pathways converge on caspase-3, resulting in the cleavage of other proteases and leading to apoptosis. In addition to caspase-dependent pathways, caspase-independent pathway is also implicated in retina apoptosis [79]. In the following sections, we discuss the potential pathways that may play a role in ER stress-associated apoptosis in various retinal diseases, such as age-related macular degeneration, glaucoma, and diabetic retinopathy (Figure 2).

### 4.1. CHOP: A Key Mediator of ER Stress-Induced Apoptosis

CHOP, also named as growth-arrest and DNA-damage-inducible gene 153 (GADD153), is a major stress-inducible proapoptotic gene in ER stress-induced apoptosis [80]. All three branches of the UPR regulate the activation of CHOP; however, ATF4 is considered as the major inducer of CHOP expression. CHOP is expressed at a very low level under physiological conditions but its expression level significantly increases in the presence of severe or persistent ER stress. Notably, the induction of CHOP well correlates with the onset of ER stress-associated apoptosis [81, 82], silencing CHOP expression protects cells against apoptosis induced by prolonged ER stress [83]. As a transcription factor, CHOP has been shown to regulate numerous pro- and antiapoptotic genes, including Bcl-2, GADD34, and TRB3 [84]. CHOP directly binds the promoter of TRB3 gene and upregulates its expression [84], which in turn inhibits AKT activation, resulting in apoptosis and cell death [85]. Intriguingly, TRB3 also regulates CHOP expression through negative feedback. Overexpressing TRB3 inhibits the transcriptional induction of CHOP while silencing TRB3 results in upregulation of CHOP under both normal and stressed conditions [86]. Treatment with PBA, a chemical chaperone that attenuates ER stress, restores AKT phosphorylation, reduces CHOP and TRB3 expression, and prevents apoptosis. These findings indicate that CHOP is a key mediator of ER stress-induced apoptosis and is tightly regulated by multiple factors, including UPR components such as ATF4 and its downstream genes such as TRB3.

### 4.2. Mitochondria and the Bcl-2 Family

Recent evidence suggests that mitochondrial dysfunction plays a role in ER stress-induced apoptosis [20, 87]. ER stress, via the UPR, also regulates a number of apoptosis-associated proteins that localize on the mitochondrial membrane, notably the members of the Bcl-2 family. These proteins are widely held as the central coordinators of mitochondria-mediated apoptotic pathways. The Bcl-2 family consists of antiapoptotic members, such as Bcl-2 and Bcl-xL, and proapoptotic proteins, such as Bax, Bak, and Bik [88]. The balance between the anti- and proapoptotic proteins is important for maintaining normal mitochondrial function as well as cell survival. Cells overexpressing Bcl-2 or deficient of Bax and Bak are resistant to ER stress-induced apoptosis [89]. Conversely, overexpressing Bax promotes cytochrome *c* release and activates apoptotic enzymes, leading to cell death [90]. BH3-only proteins, such as Bim and Bax, are proapoptotic members of the Bcl-2 protein family, playing an essential role in the initiation of programmed cell death and stress-induced apoptosis [91]. Recent studies show that both the antiapoptotic gene Bcl-2 and the proapoptotic proteins, for example, Bim and Bax, are regulated by CHOP during ER stress [87, 92]. CHOP downregulates Bcl-2 expression but upregulates Bim and promotes the translocation of Bax into the mitochondria [93]. Another BH3-only protein, p53-upregulated modulator of apoptosis (PUMA), is induced by p53 during ER stress, and PUMA-deficient cells are resistant to ER stress-elicited apoptosis. These results imply an important role of p53 and PUMA in ER stress-associated cell death [94]. In addition to mediating ER stress-driven apoptosis, the Bcl-2 family also regulates ER stress through physical interaction with ER stress sensors and UPR components. For example, both Bax and Bak have been reported to form a protein complex with IRE1*α*, which is essential for IRE1*α* activation [95]. Double knockout mice that lack Bax and Bak exhibited decreased expression of XBP1, a substrate of IRE1, and developed extensive tissue damage in the liver in response to ER stress induced by tunicamycin [95]. Thus, the mediators and pathways implicated in ER stress-related apoptosis are very complex. Nevertheless, the interdependent regulation of Bcl-2 proteins and the UPR appears to be a key event in the process of fine tuning of pro- and antiapoptotic system during ER stress.

### 4.3. Caspase-12: An ER-Resident Caspase

Caspase-12 is a member of the inflammatory group of the caspase family, localized to the ER. Moreover, it has been shown that caspase-12 is specifically activated by ER stress, including disruption of ER calcium homeostasis and accumulation of excess proteins in ER, but not by membrane- or mitochondrial-targeted apoptotic signals [96]. Mice deficient of caspase-12 are resistant to ER stress-induced apoptosis, suggesting that caspase-12 plays a critical role in this process [96]. However, the human caspase-12 gene has a single nucleotide polymorphism, which results in the production of either a truncated caspase-12 protein or a full-length protein with no enzymatic activity [97]. In human, caspase-4, a member of caspase-1 subfamily that includes caspase-12, was found localized to the ER membrane and activated specifically by ER stress-inducers [98]. Cleavage of caspase-4 was not affected by overexpression of Bcl-2, which prevents signal transduction on the mitochondria, suggesting that caspase-4 is primarily activated in ER stress-induced apoptosis [98]. Furthermore, a reduction of caspase-4 expression by small interfering RNA decreased ER stress-induced apoptosis in some cell lines, but not other ER stress-independent apoptosis [98]. Although the role of caspase-12 (caspase-4 in human) has been well established, it remains unclear how caspase-12 is activated during ER stress. Recent studies suggest that caspase-12 activation requires the IRE1 signal [99]. Upon activation by ER stress, the cytosolic domain of IRE1 recruits TNF receptor-associated factor 2 (TRAF2), which interacts with caspase-12 and induces the cleavage and activation of the enzyme [100]. In turn, activated caspase-12 cleaves procaspase-9 into active caspase-9, which further cleaves and activates caspase-3, resulting in apoptosis [101]. In addition, caspase-12 can also be activated by its downstream executioner caspase-7, indicating a possible amplification loop in the apoptotic cascades though caspase-12 [102]. Notably, in caspase-12-mediated apoptotic process, cytochrome *c* is not released from mitochondria, which suggests that cytochrome *c* is not involved in the caspase-12-dependent apoptosis [101].

### 4.4. The JNK Pathway in ER Stress-Mediated Apoptosis

The c-Jun N-terminal kinases/stress-activated protein kinase (JNK/SAPK) pathway is one of three members of the mitogen-activated protein kinase (MAPK) superfamily which also includes the ERK and the p38 MAPKinases [103]. JNK is originally identified for specifically phosphorylating the transcription factor c-jun in its N-terminal transactivation domain [103]. There are three different isoforms of JNK (JNK1, 2, and 3). Among these isoforms, JNK1 and JNK2 are ubiquitously expressed while the expression of JNK3 is tissue specific [104]. It has been reported that JNK is activated by various stress factors and contributes to apoptosis and cell death [105, 106]. Recent evidence suggests that JNK activation is also involved in ER stress-initiated apoptotic cascades [107]. For example, activation of IRE1 by ER stress recruits and activates tumor necrosis factor receptor-associated factor 2 (TRAF2), which further activates JNK [99], resulting in caspase-12 activation and apoptosis [100]. IL-1*β*, a proinflammatory cytokine, stimulates JNK activation and enhances ER stress in pancreatic epithelial cells [108]. Pretreatment with JNK inhibitor abrogates IL-1*β*-induced ER stress, indicated by phosphorylation of eIF2*α*, and increased expression of CHOP, GADD34, ATF4, and spliced XBP-1 while inhibition of ER stress does not affect JNK activation by IL-1*β* [108]. This suggests that JNK activation is required for IL-1*β*-induced ER stress. In addition, recent studies demonstrate that inhibiting JNK resulted in reduced ATF4 expression during osteoblast differentiation. JNK inhibition also alleviated Bcl-2 antagonist-induced ER stress in a lymphoma cell line [109, 110]. These findings collectively indicate a pivotal role of JNK in induction of ER stress and in mediating ER stress-induced apoptosis, which is yet to be studied in retinal cell apoptosis and retinal diseases.

### 4.5. Fas-FasL-Induced Apoptosis

The Fas death receptor belongs to the TNF receptor superfamily and is known as important inducer of apoptosis. Fas, through binding to its ligand FasL, recruits and activates the zymogen (precursor) form of cysteine protease caspases, particularly procaspase-8 and -10, which in turn activate caspase-3 and the downstream apoptotic cascades [111, 112]. Previous studies reported that Fas-FasL system is activated in diabetic retinopathy and is implicated in retinal vascular cell death in diabetic animals [113]. Treatment of retinal endothelial cells with neutrophils isolated from patients with diabetic retinopathy induced adhesion of neutrophils to endothelium and caused endothelial apoptosis [114]. Blockade of the Fas-FasL interaction prevented retinal endothelial apoptosis [114]. In an *in vivo* study, inhibiting FasL potently reduced retinal vascular endothelial cell injury, apoptosis, and blood-retinal barrier breakdown in diabetic animals [115]. Increased immunoreactivity of Fas/FasL and Fas-associated death domain (FADD) was observed in retinal glial cells and ganglion cells in rats with experimental glaucoma [116]. These findings suggest an important role of Fas-FasL system in retinal cell death. While it is widely held that binding and interaction of Fas and FasL are important for activation of the Fas signaling, recent studies suggest that Fas can also be regulated independently of FasL. Timmins and associates reported that Fas expression was induced by ER stress through a pathway involving calcium/calmodulin-dependent protein kinase IIgamma (CaMKIIgamma) and JNK [117]. In addition, activation of CaMKII by ER stress also activated STAT1, a proapoptotic signal transducer, and induced mitochondrial-dependent apoptosis, including release of mitochondrial cytochrome *c* and loss of mitochondrial membrane potential [117]. It was proposed that prolonged CHOP expression leads to the release of ER calcium stores, which increases cytosolic calcium concentration, resulting in CaMKII activation and apoptotsis. The role of CaMKII in retinal cell apoptosis remains to be elucidated.

## 5. Perspectives

Emerging evidence suggests that ER stress plays a pivotal role in retinal apoptosis and cell death. Studies in other fields have identified a number of signaling pathways that are implicated in ER stress-mediated apoptotic process. These include CHOP induction, caspase-12 activation, mitochondria dysfunction, JNK activation, Fas-FasL system and the STAT1 pathway. Blocking each of these pathways reduces or prevents ER stress-induced apoptosis to a certain extent; however, induction of an individual proapoptotic pathway may not be sufficient to induce apoptosis [117]. This suggests that prolonged ER stress may activate multiple subthreshold proapoptotic pathways, and these pathways interact and regulate each other to execute apoptosis. In addition, prolonged ER stress may also suppress the compensatory cell survival pathways induced by the UPR, such as the IFN-*β* and Akt-p38*α* pathways [117]. Recently, we demonstrated that enhancing the endogenous adaptive UPR system by preconditioning retinal cells with mild ER stress was able to reduce vascular inflammation and retinal vascular leakage [74]. Moreover, inducing molecular chaperones, for example, heat shock protein 90 (Hsp90), by antibiotics [118] prevents protein aggregation and that protects photoreceptors against retinal degeneration in a murine model of autosomal dominant retinitis pigmentosa (ADRP) [119]. In addition, overexpressing Bip/Bip in retinal photoreceptors alleviated ER stress, reduced CHOP expression, and mitigated photoreceptor apoptosis in P23H rhodopsin transgenic rats [61]. These findings support an essential role of the adaptive URP system and the URP-activated survival pathways in protecting retinal cells against apoptosis and cell death. Therefore, identifying the key proapoptotic and antiapoptotic pathways implicated in ER stress-associated apoptosis and addressing how these pathways are involved in different pathological conditions of retinal cells may offer the opportunity for developing new drugs to treat retinal diseases.
